# MECP2 Gene-Related Severe Neonatal Encephalopathy: A Rare Case in a Female Neonate

**DOI:** 10.7759/cureus.101620

**Published:** 2026-01-15

**Authors:** Risha Devi, Deeksha Gupta, Pavan K RC, Poonam Singh, Mayank Priyadarshi, Suman Chaurasia, Sriparna Basu

**Affiliations:** 1 Neonatology, All India Institute of Medical Sciences, Rishikesh, Rishikesh, IND; 2 Pediatrics, All India Institute of Medical Sciences, Rishikesh, Rishikesh, IND

**Keywords:** hypoxic ischemic encephalopathy, mecp2 gene, neonatal encephalopathy, rett syndrome, variant of unkown significance

## Abstract

Neonatal encephalopathy is a common morbidity witnessed by neonatologists globally. Though hypoxic ischemic encephalopathy (HIE) is the most common cause of neonatal encephalopathy, the absence of a specific history of hypoxia-ischemia necessitates knowledge about other diseases causing encephalopathy among neonates. These include a plethora of central nervous system, neuromuscular, vascular, metabolic, and genetic disorders. We describe a rare case of neonatal encephalopathy caused by a Methyl CpG binding protein 2 (MECP2) gene mutation in a female neonate. MECP2 mutations mostly lead to classical or variant Rett syndrome in females with a later presentation, while neonatal encephalopathy is almost exclusively reported in males. The case highlights the need for genetic testing for non-HIE causes of neonatal encephalopathy to attain the correct diagnosis.

## Introduction

Neonatal encephalopathy (NE) is a clinically descriptive term encompassing features like altered consciousness, abnormal tone, poor respiratory efforts, and seizures. Its incidence is estimated at 1-3 per 1000 live births in high-income countries and much higher in low- and middle-income countries. Hypoxic-ischemic encephalopathy (HIE) is a common but not universal cause of NE and is characterized by specific intrapartum events [1]. Other causes of NE include vascular, neuromuscular, metabolic, central nervous system malformations, and infections [1]. With rapid advancement in tools for genetic diagnoses, the proportion of cases of NE resulting from genetic causes is increasingly recognized [1].

A recent study reported 32.4% of the cases of hypoxic brain injury to have underlying genetic etiology, emphasizing the role of genetic testing in cases of NE [2]. We present a case of NE delivered at our institute in which genetic testing provided a possible explanation in the form of methyl CpG binding protein 2 (MECP2) gene mutation. The *MECP2 *gene encodes for a transcriptional regulator, methyl CpG binding protein 2, that plays a crucial role in neuronal development. *MECP2 *gene mutations have been linked with a spectrum of neurodevelopmental disorders such as Rett syndrome, X-linked intellectual disability, severe NE, Angelman’s syndrome (AS), and autism [3].

## Case presentation

A 30-year-old second gravida mother delivered a female neonate, spontaneous conception in a non-consanguineous marriage, weighing 1420 grams (small for gestational age, 1.44 centile as per Intergrowth 21^st^ size at birth charts) at 34+2 weeks of gestation. Neonate was delivered by emergency caesarean section in view of severe preeclampsia after giving injection magnesium sulfate. The antenatal period was uneventful, and antenatal scans were normal before presenting to us. The previous pregnancy was 2 years back and culminated in an early first-trimester abortion. There was no history of fever, rash, leaking or bleeding per vaginum, or gestational diabetes mellitus. She presented with features of severe preeclampsia at our institute for the first time. There was no significant family history of any neurological disorder. The neonate was born limp and apneic and required positive pressure ventilation for 2 minutes. The APGAR score at 1 and 5 minutes was 5 and 7, respectively, and the baby was shifted to the neonatal intensive care unit on continuous positive airway pressure. Cord blood gas (pH 7.36, bicarbonate 20.7 mmol/L, base deficit 3.1 mmol/L, lactate 1.6 mmol/L) was within the normal limits.

On preliminary assessment at birth, the neonate was found to be encephalopathic with marked hypotonia and reduced spontaneous movements. There was no evident dysmorphism or obvious major congenital anomaly, and head circumference was normal (30 cm, 16.12 centile as per Intergrowth 21^st^ size at birth charts). In view of poor respiratory efforts, the baby was mechanically ventilated for 46 hours of life, following which non-invasive respiratory support was progressively weaned over the next 5 days. Amplitude integrated electroencephalogram (aEEG) showed discontinuous normal voltage (minimum amplitude below 5 mcV, and maximum amplitude above 10 mcV). Ultrasound abdomen and renal and liver function tests done on day 2 were normal. The neonate remained hemodynamically stable throughout the clinical course.

The first differential for perinatal depression was considered as hypermagnesemia, as the mother received antenatal magnesium sulphate for severe preeclampsia. Baby’s serum magnesium level was also raised to 3.43 mg/dL (normal: 1.5-2.2 mg/dL). A neurosonogram done on day 1 revealed cerebral edema. At 68 hours of life, she had persistent encephalopathy and developed multiple clonic seizures, showing repeated seizure activity in aEEG, controlled with injection levetiracetam. Persistent encephalopathy with seizures in the absence of features of perinatal asphyxia warranted a work-up for an alternative diagnosis. Sepsis screen, blood culture, and cerebrospinal fluid analysis done on day 3 were within normal limits. Neurosonogram repeated on day 3 revealed ventriculomegaly (ventricular index 18 mm on the right side and 21 mm on the left side) with Grade I intraventricular hemorrhage. Subsequently, a blood profile for intrauterine infections and magnetic resonance imaging (MRI) brain were planned, and fundus examination was normal. Though encephalopathy improved over the next 1 week, hypotonia and stupor persisted. Enteral nutrition was initiated after 24 hours of life, and full enteral nutrition was achieved by day 5. The neonate was able to feed with a spoon by day 14 of life.

**Figure 1:**
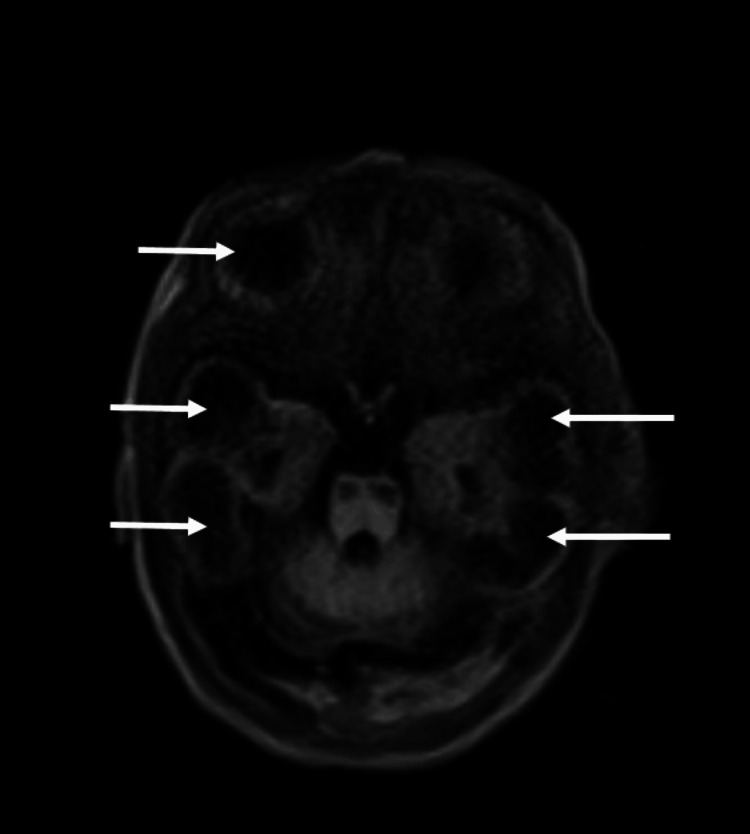
MRI brain MRI Brain (T2-axial image) showing cerebral atrophy, and multi-cystic encephalomalacia (white arrows) with cystic changes in the midbrain.

MRI brain done on day 12 revealed cerebral atrophy, cerebellar dysplasia, and multicystic encephalomalacia with cystic changes in the midbrain (Figure 1). Profile for intrauterine infections was negative, following which a next-generation clinical exome sequencing was done. It reported a single nucleotide variation in the methyl CpG binding protein 2 (MECP2) gene at exon 3 (c.1477G>A; p.Val493Met) with chromosomal location chrX: 153295838C>T as a variant of unknown significance (VUS). However, this mutation is known to have an X-linked dominant inheritance and cause *MECP2-*related severe NE and Rett syndrome. Subsequently, we planned for parental sequencing to evaluate the pathogenic role of the above mutation, but it could not be done because of financial constraints.

At discharge on day 26 of life, the neonate had hypotonia and ventriculomegaly, and seizures were controlled on a single anti-epileptic medication, and the baby was accepting full oral feeds slowly.

## Discussion

The methyl CpG binding protein 2 (*MECP2*, OMIM*300005) gene is responsible for encoding the MECP2 protein and is located on the X chromosome. This gene regulates transcription, remodels chromatin, and is involved in microRNA processing [4]. The MECP2 protein is most commonly found in the neurons, where it is responsible for their development and maturation. The most widely reported mutation of this gene causes Rett Syndrome in females and occasionally males (OMIM #312750) [5] while a wide spectrum of clinical presentation is seen in males including severe NE (OMIM #300673), mental retardation syndrome (OMIM #300055), susceptibility to autism (OMIM # 300496) and *MECP2 *duplication syndrome (OMIM # 300260) [6-8].

Severe NE associated with *MECP2 *mutations has been almost exclusively reported in males except for a few sporadic reports [9], so much so that *MECP2-*related disorders are to be considered in differential diagnosis if a male presents with NE [6]. Clinical features reported in this disease include hypotonia, breathing difficulty and apnea, seizures, microcephaly, severe developmental delay, normal birth parameters, polymicrogyria, metabolic-degenerative type of pattern, and electroencephalogram abnormalities [6,10]. It has been reported that most infants with this phenotype have a short life span of up to 2 years [11].

A large cohort study conducted by the European X-Linked Mental Retardation (XLMR) consortium included 134 male patients with severe encephalopathy and reported an *MECP2 *gene mutation incidence of up to 2% in males, while none of the females had severe NE [12]. Moreover, none of the female family members of diseased males had any symptoms, indicating that encephalopathy is rare in females. The authors attributed this to a skewing of X chromosome inactivation [12].

Another cohort study screened for *MECP2 *mutations in 78 neurologically impaired patients and reported the presence of mutations in six (7.7%) [9]. Amongst these, four were females with Rett syndrome, while one male and one female had severe NE. The female patient had a normal head circumference, hypotonia, seizures, kyphoscoliosis, and poor growth [9].

The case reported currently is unique in many ways. There is no family history of neurological impairment, and the patient is female with early onset of severe encephalopathy, seizures, and apnea - features that have been reported predominantly in males. Another finding not elucidated previously is a markedly abnormal MRI brain that may be the most severe phenotype of *MECP2 *mutation reported to date, whereas previous studies have reported mild cerebral atrophy in their cases with *MECP *mutations [13-15]. However, the findings in the present case could be attributed to antepartum hypoxia in the setting of severe preeclampsia. We agree that the identified mutation is classified as VUS, but the similarity of clinical features associated with pathogenic variants of the gene and the described case necessitates documentation of this VUS with its clinical features so that further in vitro research on animal models or computational analysis can be done with ever-increasing new reports. It would add to the expanding database for *MECP2 *mutations.

## Conclusions

To conclude, NE is caused by a wide range of etiologies besides HIE, and work-up for alternative causes is warranted if it occurs in the absence of a sentinel event or fetal growth restriction. Next-generation clinical exome sequencing is an apt tool for diagnosing rare causes as new genotype-phenotype correlations are being reported with each passing day. Subsequently, genetic counseling is important for the family to understand their neonate's condition and plan future pregnancies. Though we could not establish the pathogenic role of this mutation as a cause of NE in our case, we wish to report the occurrence of this mutation in the setting of NE because one of the manifestations of *MECP2 *gene mutations is encephalopathy. Future reports and cohort studies would educate us further about the ever-expanding phenotypic spectrum of *MECP2 *mutations.
